# Mitochondrial implications in human pregnancies with intrauterine growth restriction and associated cardiac remodelling

**DOI:** 10.1111/jcmm.14282

**Published:** 2019-04-02

**Authors:** Mariona Guitart‐Mampel, Diana L. Juarez‐Flores, Lina Youssef, Constanza Moren, Laura Garcia‐Otero, Vicente Roca‐Agujetas, Marc Catalan‐Garcia, Ingrid Gonzalez‐Casacuberta, Ester Tobias, José C. Milisenda, Josep M. Grau, Fàtima Crispi, Eduard Gratacos, Francesc Cardellach, Glòria Garrabou

**Affiliations:** ^1^ Muscle Research and Mitochondrial Function Laboratory, Faculty of Medicine and Health Sciences Internal Medicine Service‐Hospital Clínic of Barcelona, Cellex‐IDIBAPS, University of Barcelona Barcelona Spain; ^2^ CIBERER‐U722 Madrid Spain; ^3^ BCNatal—Barcelona Center for Maternal‐Fetal and Neonatal Medicine (Hospital Clínic and Hospital Sant Joan de Déu), IDIBAPS, University of Barcelona Barcelona Spain; ^4^ CIBERER‐U719 Madrid Spain

**Keywords:** bioenergetics, foetal growth, mitochondria, Sirt 3

## Abstract

Intrauterine growth restriction (IUGR) is an obstetric complication characterised by placental insufficiency and secondary cardiovascular remodelling that can lead to cardiomyopathy in adulthood. Despite its aetiology and potential therapeutics are poorly understood, bioenergetic deficits have been demonstrated in adverse foetal and cardiac development. We aimed to evaluate the role of mitochondria in human pregnancies with IUGR. In a single‐site, cross‐sectional and observational study, we included placenta and maternal peripheral and neonatal cord blood mononuclear cells (PBMC and CBMC) from 14 IUGR and 22 control pregnancies. The following mitochondrial measurements were assessed: enzymatic activities of mitochondrial respiratory chain (MRC) complexes I, II, IV, I + III and II + III, oxygen consumption (cell and complex I‐stimulated respiration), mitochondrial content (citrate synthase [CS] activity and mitochondrial DNA copy number), total ATP levels and lipid peroxidation. Sirtuin3 expression was evaluated as a potential regulator of bioenergetic imbalance. Intrauterine growth restriction placental tissue showed a significant decrease of MRC CI enzymatic activity (*P* < 0.05) and CI‐stimulated oxygen consumption (*P* < 0.05) accompanied by a significant increase of Sirtuin3/β‐actin protein levels (*P* < 0.05). Maternal PBMC and neonatal CBMC from IUGR patients presented a not significant decrease in oxygen consumption (cell and CI‐stimulated respiration) and MRC enzymatic activities (CII and CIV). Moreover, CS activity was significantly reduced in IUGR new‐borns (*P* < 0.05). Total ATP levels and lipid peroxidation were preserved in all the studied tissues. Altered mitochondrial function of IUGR is especially present at placental and neonatal level, conveying potential targets to modulate obstetric outcome through dietary interventions aimed to regulate Sirtuin3 function.

## 1 INTRODUCTION

Intrauterine growth restriction (IUGR) is a common pregnancy complication that arises when the foetus does not achieve its growth potential and affects 5%‐10% of all pregnancies.^1^, ^2^ Recent evidence demonstrated that IUGR is associated with foetal cardiovascular remodelling (CVR)^5^, ^6^ which may eventually result in a cardiovascular risk in adulthood.^7^, ^8^

However, the aetiopathology that underlies IUGR is still a matter of doubt. In many cases, IUGR is associated with placental insufficiency.^11^, ^12^ The placenta is the organ enabling nutrient and oxygen supply to the foetus.^14^, ^15^ Consequently, it plays a pivotal role for the adequate foetal development, since it requires high energy production for metabolic processes and cell growth.^16^, ^17^

Mitochondria are key organelles responsible for providing cellular energy in terms of ATP synthesised in the mitochondrial respiratory chain (MRC). Thus, mitochondria are essential for successful foetal development^19^, ^20^ and proper cardiac function.^21^, ^22^ Consequently, abnormalities in mitochondrial function and uteroplacental vessels formation have been associated with both adverse perinatal outcomes (preterm birth, IUGR, preeclampsia or stillbirth)^20^ and cardiac disease.^23^

Mitochondria are governed by nuclear effectors such as sirtuins, aimed to modulate mitochondrial disturbances depending on cellular energetic needs and dietetic habits.^24^, ^25^ For instance, there is evidence that Sirtuin3 would modulate mitochondrial respiration and attenuate reactive oxygen species (ROS) production by activating and deactivating mitochondrial target proteins through deacetylation of key lysine residues.^26^, ^27^ The emergent role of Sirtuin3 in regulating diverse pathways in mitochondrial metabolism and stress response is evidenced in a situation of nutrient restriction^29^ and also in cardiovascular disease.^24^, ^25^ Interestingly, both nutrient and CVR have been associated with IUGR.

However, there are few and quite contradictory mitochondrial studies in human IUGR pregnancies. Some have reported a mitochondrial implication in placenta by transcriptomic analysis,^30^ metabolic disarrangements in serum of IUGR infants^31^ or less mitochondrial DNA (mtDNA) in cytotrophoblast cells.^18^ Mandó et  al have also described lower MRC mRNA expression in cytotrophoblast cells but no alterations at protein level.^18^ While others found altered MRC enzymatic activities in human placental homogenate.^32^

Overall, these studies pointed out mitochondrial alterations as a potential target in IUGR but also highlighted the need for deep mitochondrial characterisation where Sirtuin3 implication may also be explored. Our group previously evidenced mitochondrial transcriptomic, ultrastructural and function alterations in the target tissue of CVR (heart) and placental insufficiency (placenta) in a rabbit model of IUGR.^33^, ^34^ To validate these findings in human pregnancies, the present work hypothesised that similar mitochondrial disarrangements would be present in human placenta.^34^ This information could generate fresh insights in disease aetiology that, in turn, could be useful to develop targeted interventions aimed to reverse this obstetric outcome. Secondly, the present study was designed to overcome target tissue limitation of CVR in humans by evaluating potential mitochondrial deficits in peripheral tissues as maternal peripheral blood mononuclear cells (PBMC) and neonatal cord blood mononuclear cells (CBMC). The benefit of finding mitochondrial abnormalities in peripheral tissues would be the development of new potential biomarkers and therapeutic targets.

## 2 EXPERIMENTAL

### 2.1 Study design

A single‐site, cross‐sectional and observational study at the Maternal‐Fetal Medicine Department of the Hospital Clinic of Barcelona (Spain) was conducted for 2 years.

### 2.2 Study population

This study included 14 IUGR pregnancies, defined as estimated birth weight <3rd percentile or, alternatively, <10th percentile in case of abnormal uterine artery Doppler or abnormal cerebroplacental ratio.^35^, ^36^ Birth weight percentile was calculated considering birth weight, weeks of gestation and neonatal gender. Despite final IUGR diagnostic is confirmed at delivery, all potential IUGR pregnancies were monitored by Doppler every week during gestation. In parallel, 22 uncomplicated pregnancies were considered as the control group.^11^

The inclusion criteria were: >18 years, singleton pregnancies, >22 weeks of gestation and no tobacco consumption in both IUGR and control pregnancies. Pregnant women taking potentially toxic drugs for mitochondria and with familial history of mitochondrial disease were excluded.

The study was approved by the Ethical Committee of our hospital (2013/8246) and followed the Declaration of Helsinki guidelines. All participants provided a written informed consent.

### 2.3 Sample collection and processing

At delivery, placental samples were weighted and, after discarding blood residuals, a full thickness section (from both maternal and foetal side) was obtained and processed as follows: 500 mg were homogenised (Caframo technologies, Ontario, Canada) with 10% BSA‐SolutionA to isolate fresh mitochondria and immediately perform ex vivo oxygen consumption assays.^37^ The remaining tissue was immediately cryopreserved at −80°C and further homogenised at 5% (w/v) in Mannitol for the rest of mitochondrial analysis.

Additionally at delivery, 10‐20 mL of maternal peripheral blood and neonatal cord blood were collected in ethylenediaminetetraacetic acid‐tubes to isolate plasma, PBMC and CBMC by a Ficoll gradient.^38^ One aliquot of each sample was maintained in fresh conditions to assess ex vivo oxygen consumption. The remaining aliquots were stored at −80°C for mitochondrial analysis.

The usefulness of mononuclear cells for the study of mitochondrial dysfunction has been previously validated. It offers an accessible and non‐invasive approach and the possibility to find new potential biomarkers for diagnosis and prognosis.^39^, ^40^

Protein content was measured through the bicinchoninic acid colorimetric assay following manufacturer's instructions (Thermo Scientific, Waltham, MA) to normalise experimental measurements.

### 2.4 BNP levels in cord blood

As a reliable marker of neonatal cardiac remodelling,^42^ brain natriuretic peptide (BNP) levels were measured in neonatal plasma by the CORE laboratory of our Hospital using an Advia Centaur XP.^43^

### 2.5 Mitochondrial oxygen consumption

Based on previous transcriptomic results pointing out MRC complex I (CI) deregulation,^33^ oxygen consumption was measured through the stimulation of CI. Briefly, isolated placental mitochondria, PBMC and CBMC were used in fresh conditions to determine oxygen consumption by polarography (Hansatech Instruments, Pentney, UK) in a ‘respiration medium’ (0.3 mol/L Mannitol, 10 mmol/L KCl, 5 mmol/L MgCl_2_·6H_2_O and 10 mmol/L potassic phosphate).^44^ Cellular oxygen consumption for endogenous substrates (abbreviated as Cellox) was measured in intact PBMC and CBMC. Digitonin was then used to permeabilise blood cells. Manual titration of substrates for CI stimulation (1 mol/L glutamate and 0.5 mol/L malate or 0.25 mol/L pyruvate and 0.5 mol/L malate; referred as GM oxidation, GMox and PM oxidation, PMox) was performed using Hamilton syringes (Hamilton Company, Reno, NV). Respiratory quality controls were added into the assay by assessing drug response to phosphate acceptors (50 mmol/L Adenine diphosphate), uncouplers (0.25 µmol/L CCCP) and respiratory inhibitors (0.2 mmol/L antimycin a). Data were recorded using O_2_view Software.

The results were expressed as picomoles of consumed oxygen per second and milligram of protein (pmol O_2_/s mg prot.).

### 2.6 Mitochondria respiratory chain enzymatic activities and mitochondrial content

#### 2.6.1 Enzymatic activities of MRC and citrate synthase

The enzymatic activities of MRC complexes I, II, IV, I + III and II + III (CI, CII, CIV, CI + III and CII + III) were measured spectrophotometrically in placenta according to national standardised methods.^45^ Following the same protocols, the measurement of enzymatic activities of MRC in maternal PBMC and neonatal CBMC was restricted to CII and CIV based on previous results of mitochondrial function obtained from the animal model.^34^

Citrate synthase (CS) activity was also measured spectrophotometrically,^41^, ^45^ as an enzyme belonging to the tricarboxylic acid (TCA) cycle, widely used as a reliable marker of mitochondrial content.^46^

Absorbance changes along time were monitored in a HITACHI‐U2900 spectrophotometer using the UV‐Solution software 2.2 and were expressed as nanomoles of consumed substrate or generated product per minute and milligram of protein (nmol/min·mg protein).

#### 2.6.2 Mitochondrial DNA levels

Alternative measurement to determine mitochondrial content was performed by analysing mtDNA copy number. Thus, total DNA was phenol‐chloroform‐extracted from neonatal CBMC. Multiplex qPCR 7500 Real Time PCR System from Applied Biosystems (Foster City, CA) was used.^47^

Briefly, quantification of the mitochondrial 12S ribosomal RNA (mt12SrRNA) gene and the constitutive nuclear RNAseP gene was performed and results were expressed as the mt12SrRNA/nRNAseP ratio.

### 2.7 Total cellular ATP levels

Cellular ATP levels were quantified in each tissue using the Luminescent ATP Detection Assay Kit (Abcam, Cambridge, UK). The results were expressed as picomolar of ATP per milligram of protein (pmol ATP/mg protein).

### 2.8 Lipid peroxidation (oxidative damage)

Lipid peroxidation was measured as an indicator of oxidative damage in to lipid membranes using the BIOXYTECH‐LPO‐586™ assay by the spectrophotometric measurement of malondialdehyde (MDA) and 4‐hydroxyalkenal (HAE) levels (Oxis International Inc, Los Angeles, CA, USA). The results were expressed as micromolar of MDA and HAE per milligram of protein (μmol/L MDA + HAE/mg protein).

### 2.9 Expression of Sirtuin3 in placenta

The protein content of Sirtuin3, a sensor of mitochondrial and metabolic balance, was determined by Western blot. Forty µg of total protein placental homogenate were separated using 7/13% SDS‐PAGE and transferred into nitrocellulose membranes (iBlot Gel Transfer Stacks; Life Technologies, Waltham, MA, USA). The membranes were hybridised with anti‐Sirtuin3 (44 KD; 1:250; Merck Millipore, Burlington, MA, USA) overnight and at 4°C. Sirtuin3 protein expression was normalised by β‐actin protein (47 KD; 1:30 000; Sigma‐Aldrich, St. Louis, MO). The ImageQuantLD program was used to quantify chemiluminescence and the results were expressed as the Sirtuin3/β‐actin ratio.

### 2.10 Statistical analysis

Statistics were performed using the ‘IBM SPSS Statistics 20’ software. Clinical and experimental results were filtered once for outliers and expressed as percentage, means ± SEM or percentage of increase/decrease of IUGR cases compared to controls. Comparisons at placental, maternal or neonatal level were always done between cases and controls (IUGR versus control pregnancies). Nonparametric tests were used to determine: case‐control differences (Mann‐Whitney independent sample analysis or odds ratio by Fisher's exact test) and parameter correlation (Spearman's rank coefficient). Significance was set at *P* < 0.05.

## 3 RESULTS

### 3.1 Clinical parameters

Table 1 shows sociodemographic characteristics and perinatal outcomes of study groups. No differences were found between groups regarding maternal age, mode of delivery, new‐born sex or pH umbilical artery cord blood.

**Table 1:** Sociodemographic characteristics and perinatal outcomes of the study groups

Parameter	Control N = 22	IUGR N = 14	*P* value
Maternal age (y)	33.82 ± 1.12	34.43 ± 1.34	NS
Weeks of gestation at delivery	39.47 ± 0.20	35.42 ± 1.03	<0.001
Mode of delivery	20 caesarean section (91%) Two vaginal (9%)	14 caesarean section (100%) 0 vaginal (0%)	NS OR [95% CI] = 0.28 [0.01‐6.34]
Birth weight (g)	3440.27 ± 87.59	1742.29 ± 171.31	<0.001
Birth weight percentile	58.32 ± 5.94	0.64 ± 0.23	<0.001
Placental weight (g)	566.67 ± 118.93	329.64 ± 24.26	<0.05
New‐born sex	45% women 55% men	54% women 46% men	NS OR [95% CI] = 0.70 [0.17‐2.85]
pH umbilical artery cord blood	7.26 ± 0.01	7.24 ± 0.03	NS
Apgar 5ʹ	21 normal (95%) One abnormal (5%)	Nine normal (64%) Five abnormal (36%)	<0.05 OR [95% CI] = 11.67 [1.19‐114.65]
Preeclampsia	0 (0%)	4 (28.6%)	<0.05 OR [95% CI] = 0.05 [0.00‐1.06]
Cord blood BNP levels (pg/mL)	26.84 ± 3.03	85.68 ± 26.28	<0.05

Values are presented as mean ± SEM or percentage of positive cases within the study cohort. Case‐control differences were sought by non‐parametric statistical analysis.

BNP, brain natriuretic peptide; CI, confidence interval; g, grams; IUGR, intrauterine growth restriction; N, sample size; NS, not significant; OR, odds ratio; y, years.

**Figure 1:**
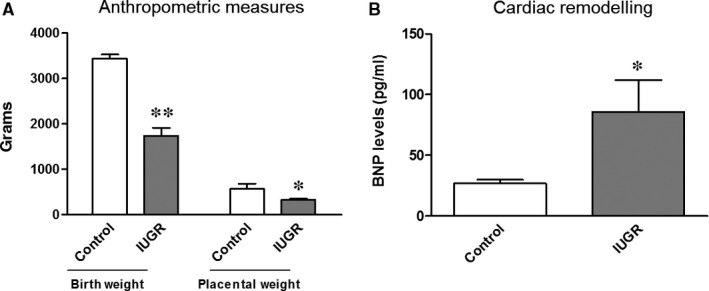
A, Anthropometric measures of the study groups. Birth weight is significantly reduced in new‐borns from IUGR pregnancies (grey bar) compared to controls (empty bar), as well as placental weight. B, Cardiac remodelling in new‐borns from IUGR pregnancies. BNP levels were significantly increased in neonatal plasma from IUGR pregnancies (grey bars) with respect to controls (empty bars). Results are expressed as mean ± SEM. Mann‐Whitney tests were used to seek for statistical analysis between groups. BNP, brain natriuretic peptide; IUGR, intrauterine growth restriction; ^**^ *P* < 0.001; ^*^ *P* < 0.05

As expected, IUGR pregnancies presented abnormal 5‐minute Apgar score (*P* < 0.05; Table 1) and an earlier gestational age at delivery compared to controls (*P* < 0.001; Table 1) because our clinical protocol for IUGR indicate induction of delivery about 37 weeks of gestation. Additionally, birth weight, birth weight percentile and placental weight were significantly decreased in IUGR cases with respect to controls (*P* < 0.001; *P* < 0.001 and *P* < 0.05, respectively; Figure 1A; Table 1). Moreover, IUGR group showed higher prevalence of preeclampsia (*P* < 0.05; Table 1).

Brain natriuretic peptide levels were significantly increased by 219.23 ± 97.91% in IUGR neonatal plasma compared to controls (*P* < 0.05; Figure 1B; Table 1).

### 3.2 Mitochondrial study in placenta

#### 3.2.1 Mitochondrial oxygen consumption

**Figure 2:**
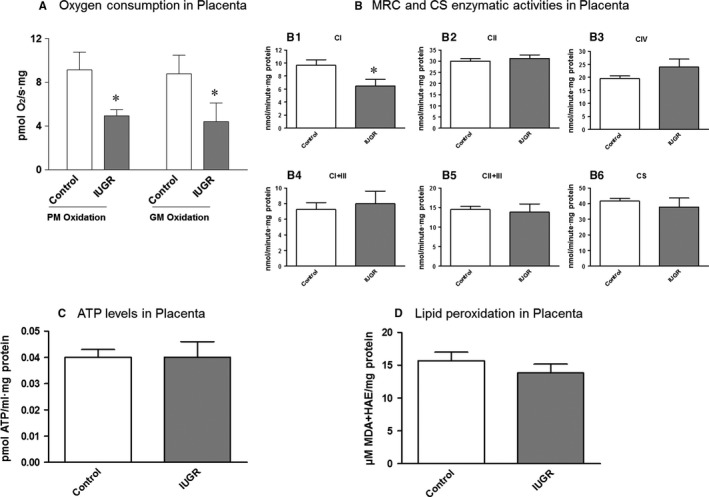
A, Oxygen consumption in placental mitochondria of the study groups. A significant decrease in MRC Complex I‐stimulated oxygen consumption (both PM and GM Oxidation) was observed in the IUGR cohort (grey bars) compared to controls (empty bars). B, Enzymatic activities of the complexes of the MRC and CS in placental tissue of the study groups. A significant decreased was observed of complex I activity in placenta from IUGR pregnancies (B1: grey bars) while other complexes (B2, B3, B4, B5) and also CS (B6) activity remained conserved. C, Total ATP levels in placental tissue of the study groups. No significant differences were observed between IUGR pregnancies (grey bars) and controls (empty bars). D, Lipid peroxidation as an indicator of oxidative damage in placental tissue of the study groups. No significant differences were evidenced in placental tissue between IUGR pregnancies (grey bars) and controls (empty bars). Results are expressed as mean ± SEM and Mann‐Whitney tests were used to seek for statistical analysis between groups. ATP, adenosine triphosphate; CI, complex I; CII, complex II; CIV, complex IV; CI + III, complex I + III; CII + III, complex II + III; CS, citrate synthase; GM oxidation, glutamate/malate oxidation; HAE, 4‐hydroxyalkenal; IUGR, intrauterine growth restriction; MDA, malondialdehyde; PM oxidation, pyruvate/malate oxidation; MRC, mitochondrial respiratory chain; ^*^ *P* < 0.05

Significant decreases of 46.12 ± 5.79% and 49.71 ± 19.54% in CI‐stimulated oxygen consumption (PMox and GMox, respectively) were observed in placental mitochondria from IUGR pregnancies compared to controls (*P* < 0.05; Figure 2A; Table S1).

#### 3.2.2 Mitochondria respiratory chain enzymatic activities and mitochondrial content

Mitochondria respiratory chain CI enzymatic activity was also significantly decreased in placenta from IUGR pregnancies compared to controls (−32.95 ± 10.36%; *P* < 0.05; Figure 2B; Table S1), despite other MRC complexes (CII, CIV, CI + III and CII + III) were preserved (Figure 2B; Table S1).

Citrate synthase activity was conserved in placenta between groups (Figure 2B; Table S1).

#### 3.2.3 Total cellular ATP levels

No remarkable differences were observed in ATP content between groups (Figure 2C; Table S1).

#### 3.2.4 Lipid peroxidation (oxidative damage)

No relevant changes were observed in lipid peroxidation between groups (Figure 2D; Table S1).

#### 3.2.5 Sirtuin3 protein expression

**Figure 3:**
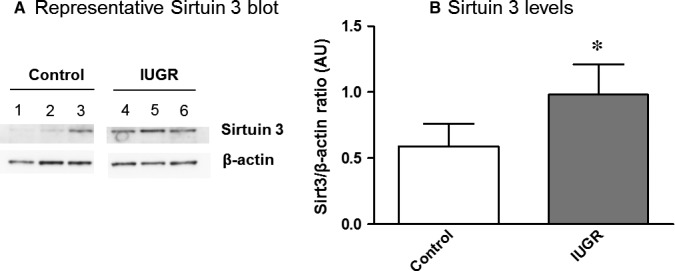
Sirtuin3 protein levels in placenta of the study groups. A, A representative Western Blot of Sirtuin3 protein expression in human placenta is shown in both controls (1‐3) and IUGR pregnancies (4‐6). β‐actin was used as the loading control. B, Graph showing the significant increase of Sirtuin3 levels (Sirt3/β‐actin ratio) in IUGR pregnancies (grey bars) compared with controls (empty bars). Results are expressed as mean ± SEM. Mann‐Whitney tests were used to seek for statistical analysis between groups. AU, Arbitrary units; IUGR, Intrauterine growth restriction; Sirt3, Sirtuin3; *P < 0.05

A significant 117.78±51.11% (according to Table S1) increase of Sirtuin3/β‐actin protein expression was observed in IUGR placenta with respect to controls (*P* < 0.05; Figure 3A,B; Table S1).

### 3.3 Mitochondrial study in maternal and neonatal mononuclear cells

#### 3.3.1 Mitochondrial oxygen consumption

**Figure 4:**
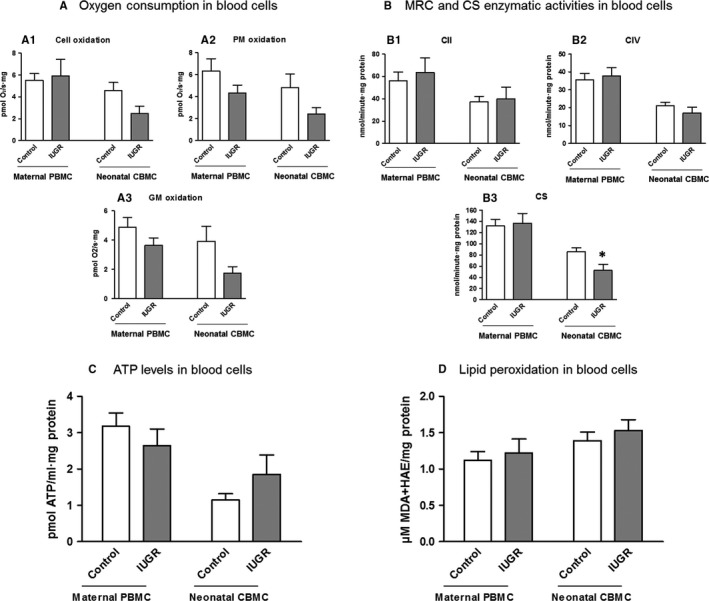
A, Oxygen consumption in maternal and neonatal blood cells of the study groups. Maternal PBMC from IUGR pregnant women (grey bars) presented conserved cellular oxygen consumption (A1) and trends to decrease of oxygen consumption stimulated for CI (A2‐3) compared to controls (empty bars). Despite not reaching statistical significance, neonatal CBMC from IUGR new‐borns (grey bars) presented a tendency to decrease of both cellular and CI‐stimulated oxygen consumption (A1‐3) compared to controls (empty bars). Additionally, IUGR new‐borns presented higher oxygen consumption deficiencies compared to mothers. B, Enzymatic activities of the complexes of the MRC and CS in maternal and neonatal blood cells of the study groups. IUGR cohort is presented as grey bars and controls as empty bars. No remarkable differences were evidenced in maternal PBMC. However, a significant decrease of CS activity was found in neonatal CBMC (B3). C, Total ATP levels in maternal and neonatal blood cells of the study groups. No significant differences were observed either in maternal PBMC or in neonatal CBMC between IUGR pregnancies (grey bars) and controls (empty bars). D, Lipid peroxidation as an indicator of oxidative damage in maternal and neonatal blood cells of the study groups. No significant differences were evidenced either in maternal PBMC and neonatal CBMC between IUGR pregnancies (grey bars) and controls (empty bars). Results are expressed as a percentage of increase or decrease with respect to controls ± SEM (A,B) and as mean ± SEM (C,D). Mann‐Whitney tests were used to seek for statistical analysis between groups. ATP, adenosine triphosphate; CBMC, cord blood mononuclear cells; cell oxidation, cellular endogen oxidation (without substrates); CI, complex I activity; CII, complex II activity; CIV, complex IV activity; CI + III, complex I + III activity; CII + III, complex II + III activity; CS, citrate synthase activity; GM oxidation, glutamate and malate oxidation); HAE, 4‐hydroxyalkenal; IUGR, intrauterine growth restriction; MDA, malondialdehyde; MRC, mitochondrial respiratory chain; PBMC, peripheral blood mononuclear cells; PM oxidation, pyruvate and malate oxidation; ^*^ *P* < 0.05

Peripheral blood mononuclear cells from IUGR pregnant women presented conserved cellular oxygen consumption (p = NS; Figure 4A; Table S2) and trends to decrease of CI‐stimulated oxygen consumption compared to controls (PMox: −31.55 ± 11.36% and GMox: −25.00 ± 10.45%; p = NS; Figure 4A; Table S2).

Despite not reaching statistical significance, CBMC from IUGR new‐borns presented a tendency to decrease of cellular and CI‐stimulated oxygen consumption compared to controls (Cellox: 45.63 ± 14.19%; PMox: −49.90 ± 11.39; GMox: –55.73 ± 11.45; all p = NS; Figure 4A; Table S3). Noticeably, IUGR new‐borns presented greater mitochondrial deficits compared to mothers.

#### 3.3.2 Mitochondria respiratory chain enzymatic activities and mitochondrial content

No differences in MRC CII and CIV enzymatic activities were observed in maternal PBMC or in neonatal CBMC (Figure 4B; Tables S2 and S3) between IUGR cases and controls.

Moreover, despite conserved CS activity in PBMC from IUGR pregnant women (Figure 4B; Table S2), there was a significant 39.19 ± 12.61% decrease in CBMC from IUGR new‐borns compared to controls (*P* < 0.05; Figure 4B; Table S3).

In order to elucidate if the decrease of CS in CBMC from IUGR new‐borns was due to abnormalities in TCA cycle or in mitochondrial content, we measured alternative markers of mitochondrial mass such as levels of mtDNA that resulted unaltered between IUGR and control groups (Table S3).

#### 3.3.3 Total cellular ATP levels

No differences in ATP content were observed in IUGR group compared to controls (Figure 4C; Tables S2 and S3).

#### 3.3.4 Lipid peroxidation (oxidative damage)

No changes were observed in lipid peroxidation between IUGR and control groups (Figure 4D; Tables S2 and S3).

### 3.4 Associations between clinical data and experimental results

**Figure 5:**
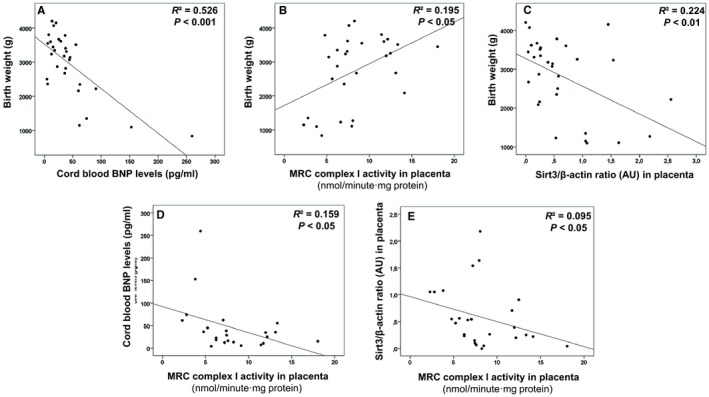
Association between clinical data and experimental results from pregnancies complicated by IUGR and controls. Decreased birth weight characteristic of IUGR is associated to increased BNP levels (as sign of cardiovascular remodelling) (A), decreased MRC CI in placenta (B) and enhanced Sirtuin3 protein expression (C). Additionally, impairment in MRC CI in placenta was directly associated to increased neonatal BNP levels (D) and placental Sirtuin3 protein expression (E), demonstrating the strong association among all these parameters. Spearman Rho tests were used to seek for statistical analysis. AU, Arbitrary units; BNP, Brain natriuretic peptide; CBMC, Cord blood mononuclear cells; CI, MRC complex I; g, grams; IUGR, intrauterine growth restriction; MRC, Mitochondrial respiratory chain; Sirt3, Sirtuin3

Birth weight, as well as placental weight, were negatively correlated with BNP levels, confirming the association of IUGR with CVR (*P* < 0.001 and *P* < 0.01, respectively; Figure 5A; Table S4). Secondly, birth weight was also positively correlated with CI‐stimulated oxygen consumption (PMox and GMox *P* < 0.05; Table S4) and CI enzymatic activity (*P* < 0.05; Figure 5B; Table S4) in placenta, suggesting that a proper new‐born weight promotes optimal MRC CI function in placenta, or more likely, that efficient placental CI function is required to reach a proper birth weight. Additionally, birth weight was negatively correlated with placental Sirtuin3 levels (*P* < 0.05; Figure 5C; Table S4), suggesting the adaptation mechanism of Sirtuin3 up regulation in response to IUGR.

**Figure 6:**
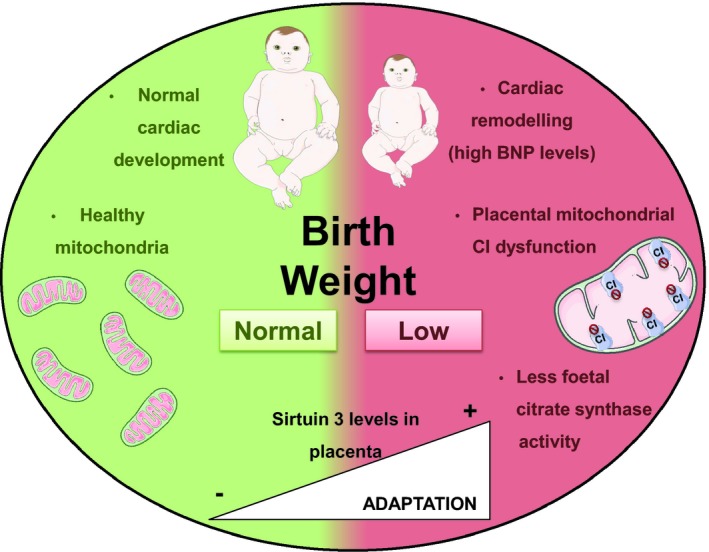
Mitochondrial and cardiac features of human pregnancies in two contexts, complicated by intrauterine growth restriction (right side in red) and with no apparent obstetric problems (left side in green). In IUGR new‐borns, low birth weight was associated to a higher levels of BNP in new‐borns (indicating a potential cardiac remodelling), lower mitochondrial complex I function in placenta and decreased neonatal citrate synthase activity (representative of metabolic mitochondrial activity). In this context, there was an increase of placental protein Sirtuin3 levels, probably as a potential adaptation mechanism aimed to modulate the adverse mitochondrial phenotype reported in this obstetric complication. On the other hand, all these mitochondrial parameters were found within normal ranges in healthy children with no apparent IUGR. BNP, brain natriuretic peptide; CI, mitochondrial respiratory chain complex I; IUGR, intrauterine growth restriction

Brain natriuretic peptide levels were also negatively correlated with CI enzymatic activity in placenta (*P* < 0.01; Figure 5D; Table S4) which additionally was negatively correlated with placental Sirtuin3 levels (*P* < 0.05; Figure 5E; Table S4). Those associations suggest that CVR is characterised by CI impairment and adaptive increase of Sirtuin3 levels in the placenta (Figure 6).

Moreover, maternal and neonatal CI‐stimulated oxygen consumption were positively correlated (GMox; *P* < 0.05; Table S4), demonstrating the strong dependence of neonatal metabolism on maternal status. Additionally, neonatal oxygen consumption positively correlated with neonatal CS activity (Cell oxidation: *P* < 0.05; PM oxidation: *P* < 0.05; and GM oxidation: *P* ≤ 0.001; Table S4), suggesting the dependence of a proper mitochondrial function in TCA activity. Finally, this neonatal oxygen consumption also positively correlated with placental CI‐stimulated oxygen consumption (*P* < 0.05; Table S4), pointing out the dependence of neonatal health on accurate placental function.

## 4 DISCUSSION

The molecular basis of IUGR and CVR is still a matter of doubt, but major concerns are raised due to the high prevalence of this obstetric problem and the putative consequences in adulthood. Previous experimental studies pointed out that mitochondrial deficits play a relevant role in IUGR and associated CVR.^33^, ^34^ However, few and contrasting studies were found in the literature investigating mitochondrial alterations in human pregnancies, pointing out the need for a wider study of mitochondrial involvement in patients with this obstetric complication.

Human mitochondrial function alterations in IUGR have been previously demonstrated in placenta, specifically in isolated mitochondria or derived cultured cells.^18^, ^30^, ^32^ Besides, only scarce data studying mitochondrial function in neonatal CBMC have been published so far.^48^ Herein, we present a wide characterisation of mitochondrial function in human placenta together with simultaneous studies in maternal PBMC and neonatal CBMC.

Our results provided evidence of mitochondrial imbalance in placenta from IUGR pregnancies focused on CI‐stimulated oxygen consumption and enzymatic activity (Figure 6). This significant CI deficiency was previously described by our group at transcriptional level in heart tissue of an IUGR animal model^33^ and also by Beyramzadeh et  al in placenta from high risk pregnancies.^32^ Conversely to our findings, Mandó et  al observed higher oxygen consumption stimulated either through CI + CII or CIV in placenta from IUGR cases.^18^ Such discrepancies may be due to differential stimulation of specific MRC complexes and the presence of different cell linages (cytotrophoblasts and syncytiotrophoblasts) that have multiple functions, different mitochondria and also respond differently to stimulus.^49^ In accordance with placental MRC CI impairment in the studied patients, placental Sirtuin3 protein expression significantly increased, probably, as an adaptation mechanism to modulate the adverse mitochondrial phenotype.

This mitochondrial imbalance was also evident in CBMC of IUGR new‐borns through the significant decrease of CS activity, not described so far. Citrate synthase is a reliable marker of mitochondrial content,^46^ but it is also an enzyme participating in the TCA cycle. As parallel measurement of alternative markers of mitochondrial content (mtDNA levels) yielded to results suggestive of preserved mitochondrial mass, we concluded that alterations in CS activity would be mainly related to new‐born metabolic imbalance of TCA cycle. Previous studies in obstetric complications associated with placental insufficiency such as IUGR or preeclampsia, showed contrasting results for mitochondrial content.^18^, ^50^, ^51^ Noticeably, in this study, mitochondrial content was maintained also in placenta and in maternal PBMC.

Maternal PBMC were neither affected for mitochondrial function alterations, reinforcing both the placenta and the new‐born as the main targets of this obstetric complication.

Remarkably, in the present study, BNP levels were significantly higher in IUGR new‐borns, confirming the neonatal CVR previously reported by our group and others.^42^, ^54^, ^55^ Additionally, birth weight was inversely associated with BNP levels confirming the strong association between IUGR and CVR; moreover, birth weight was also directly correlated to placental CI activity and inversely correlated with placental Sirtuin3 expression, thus reinforcing mitochondrial implication in this obstetric complication.

In regard to Sirtuin3, its expression has been associated to increased bioenergetic demands,^24^, ^25^ often in the context of cardiovascular disease, acting as a protective mechanism in front of different stimulus, such as mitochondrial function and oxidative damage.^29^, ^56^ This molecule is becoming of high interest as a potential target to overcome metabolic disarrangements. Additionally, it can be modulated by diet,^57^ emerging as a promising intervention for obstetric complications in which pharmacological interventions are highly discouraged. In the present study, we hypothesised that CI deficiency in placenta may promote the expression of nuclear effectors such as Sirtuin3 to compensate for depressed mitochondrial function, as we previously observed in an IUGR rabbit model.^33^ To our knowledge, this is the first study suggesting up‐regulation of Sirtuin3 in human IUGR pregnancies, suggesting its usefulness as a therapeutic target.

On the other hand, maternofoetal correlations were observed in mitochondrial parameters, confirming the dependence of neonatal bioenergetics in maternal health status.

Our results showed preserved total ATP levels either in placental tissue, maternal PBMC or neonatal CBMC, suggesting a potential switch from aerobic to faster anaerobic metabolism to preserve ATP supply.^58^ Similarly, we found no differences in lipid peroxidation, as an indicator of oxidative stress, between groups, in any tissue. Previous studies in IUGR reported controversial results in oxidative damage^49^, ^59^, ^60^ but in other pregnancy complications caused by placental insufficiency, such as preeclampsia, it is described an induction of oxidative stress in the placenta and maternal blood,^59^ as well as in certain risk pregnancies depending on the type of delivery (specially vaginal delivery because of intermittent perfusion of intervillous space of the placenta during uterine contractions).^61^ In our cohort of IUGR pregnancies, we reduced preeclampsia comorbidity and vaginal delivery to avoid potential confounders. Whether preserved oxidative damage in our sample is linked to deficient oxygen supply due to placental insufficiency and consequent mitochondrial decreased activity,^62^ the presence of antioxidant defences or other compensatory mechanisms such as Sirtuin3, is still a matter of doubt. Some limitations and technical considerations should be acknowledged in our study. First, IUGR is known to be a multifactorial obstetric condition where many pathways and aetiologies could finally lead to a unique phenotype. Further studies are warranted to better clarify the functional consequences of the decrease of placental MRC CI function and neonatal TCA activity in order to elucidate whether such a deficiency is a consequence or the cause of this obstetric complication. Additionally, larger sample size cohort studies should be used to strengthen statistical findings, in which additional measurements may ideally be studied to investigate mechanistic pathways, as well as the potential contribution of the different cell types of affected tissues in the context of IUGR (as lymphocytes and monocytes in PBMC or syncytiotrophoblast and cytotrophoblast in placenta). For instance, we cannot dismiss that cell composition and type may change in pathological conditions, thus conditioning IUGR or potential blood contamination in placental tissue. Similarly, we cannot exclude that the difference in gestational age at delivery between cases and controls could have influenced the results. This limitation is difficult to overcome as most clinical protocols indicate finalisation of gestation in IUGR with signs of placental insufficiency at 37‐38 weeks. Finally, several potential confounders influencing mitochondrial and metabolism regulation (such as vaginal delivery, preeclampsia prevalence, maternal diet and lifestyle) were not considered in this study and may play a role in observed results. Despite these considerations, the consistency of our results, as well as the different associations found between clinical parameters and experimental findings in human pregnancies, or the similarities between human findings and experimental model results,^33^ strengthen the validity of the present findings.

In conclusion, IUGR is associated with depressed mitochondrial function, especially at placental and neonatal level. Placental insufficiency seems to directly affect birth weight, CVR and mitochondria, highlighting the necessity to focus therapeutic efforts on those targets, such as dietary interventions aimed to regulate Sirtuin3.

## CONFLICT OF INTEREST

Authors report no conflict of interest.

## AUTHOR CONTRIBUTION

GG and FCa obtained funds to support the study. GG conceived the study and organised the project in collaboration with FCa, FCr, EG and MG. LY and LG‐O were responsible for the diagnosis and inclusion of all patients and collected clinical data together with DLJ‐F. All of the experimental, data collection and statistical analysis was closely supervised by GG, CM and MG‐M. MG‐M, with help from VR‐A, MC‐G, ET and IG‐C performed all the experimental procedure to evaluate mitochondrial function. Concretely, MG‐M and ET were in charge of processing all samples in the laboratory. MG‐M, together with MC‐G and IG‐C, performed in vivo measurement of oxygen consumption of each sample. The remaining molecular measurements were performed by MG‐M with the support of VR‐A. The first draft of this manuscript was written by MG‐M, and GG, FC, FC, LY, LG‐O, DLJ‐F, JCM and JMG profoundly reviewed and critiqued the manuscript, adding concepts of high relevance.
