# Supplementary material for: Mitochondrial implications in human pregnancies with intrauterine growth restriction and associated cardiac remodelling
Source: J Cell Mol Med. 2019 Apr 2;23(6):3962–73. doi: 10.1111/jcmm.14282 (PMC6533501; doi:10.1111/jcmm.14282)
Supplement: Supplementary file 1 [file JCMM-23-3962-s001.docx]

**Table S1.** **Experimental data in human placental tissue of study groups.**

| ***Mitochondrial parameters in PLACENTA*** | ***Control***  ***N = 22*** | ***IUGR***  ***N = 14*** | ***% of change*** | ***P value*** |
| --- | --- | --- | --- | --- |
| **Complex I** (nmol/minute·mg protein) | 9.65±0.83 | 6.47±1.00 | -32.95±10.36 | <0.05 |
| **Complex I relative to CS activity** (nmol/minute·mg protein) | 0.21±0.03 | 0.20±0.04 | -4.75±19.05 | NS |
| **Complex II** (nmol/minute·mg protein) | 30.06±1.06 | 31.22±1.56 | +3.86±3.16 | NS |
| **Complex II relative to CS activity** (nmol/minute·mg protein) | 0.75±0.03 | 0.96±0.13 | +28.00±17.33 | NS |
| **Complex IV** (nmol/minute·mg protein) | 19.52±1.04 | 23.93±3.07 | +22.59±15.73 | NS |
| **Complex IV relative to CS activity** (nmol/minute·mg protein) | 0.50±0.03 | 0.69±0.09 | +38.00±18.00 | <0.05 |
| **Complex I+III** (nmol/minute·mg protein) | 7.25±0.88 | 7.99±1.62 | +10.21±22.34 | NS |
| **Complex I+III relative to CS activity** (nmol/minute·mg protein) | 0.18±0.02 | 0.20±0.03 | +11.11±16.67 | NS |
| **Complex II+III** (nmol/minute·mg protein) | 14.50±0.77 | 13.79±2.14 | -4.90±14.76 | NS |
| **Complex II+III relative to CS activity** (nmol/minute·mg protein) | 0.35±0.01 | 0.41±0.04 | +17.14±11.43 | NS |
| **Citrate Synthase** (nmol/minute·mg protein) | 41.58±1.74 | 37.75±5.88 | -9.21±14.14 | NS |
| **PM oxidation** (pmol O2/s·mg) | 9.15±1.59 | 4.93±0.53 | -46.12±5.79 | <0.05 |
| **GM oxidation** (pmol O2/s·mg) | 8.75±1.71 | 4.40±1.71 | -49.71±19.54 | <0.05 |
| **ATP levels** (pmol ATP/mg protein) | 0.04±0.00 | 0.04±0.01 | 0.00±15.00 | NS |
| **Lipid peroxidation** (μM MDA+HAE/mg protein) | 15.67±1.32 | 13.82±1.34 | -11.81±8.55 | NS |
| **Sirtuin3/β-actin** (AU) | 0.45±0.10 | 0.98±0.23 | +117.78±51.11 | <0.05 |

Values are presented as mean ± standard error of the mean and as a percentage of increase or decrease ± standard error of the mean. Case-control differences were sought by non-parametric statistical analysis.

ATP: adenosine triphosphate; AU: arbitrary units; CS: citrate synthase; GM oxidation: glutamate and malate oxidation; HAE: 4-hydroxyalkenal; IUGR: intrauterine growth restriction; MDA: malondialdehyde; NS: not significant; O_2_: oxygen; PM oxidation: pyruvate and malate oxidation.
