# Supplementary material for: Mitochondrial implications in human pregnancies with intrauterine growth restriction and associated cardiac remodelling
Source: J Cell Mol Med. 2019 Apr 2;23(6):3962–73. doi: 10.1111/jcmm.14282 (PMC6533501; doi:10.1111/jcmm.14282)
Supplement: Supplementary file 2 [file JCMM-23-3962-s002.docx]

**Table S2.** **Experimental data in maternal peripheral blood mononuclear cells of study groups.**

| ***Mitochondrial parameters in Maternal PBMC*** | ***Control***  ***N = 22*** | ***IUGR***  ***N = 14*** | ***% of change*** | ***P value*** |
| --- | --- | --- | --- | --- |
| **Complex II** (nmol/minute·mg protein) | 56.18±7.63 | 63.60±13.13 | +13.21±23.37 | NS |
| **Complex II relative to CS activity** (nmol/minute·mg protein) | 0.38±0.03 | 0.39±0.02 | +2.63±5.26 | NS |
| **Complex IV** (nmol/minute·mg protein) | 35.73±3.55 | 37.82±4.56 | +5.85±12.76 | NS |
| **Complex IV relative to CS activity** (nmol/minute·mg protein) | 0.27±0.03 | 0.28±0.04 | +3.70±14.81 | NS |
| **Citrate Synthase** (nmol/minute·mg protein) | 132.38±11.53 | 137.00±17.58 | +3.49±13.28 | NS |
| **Cell oxidation** (pmol O2/s·mg) | 5.50±0.63 | 5.90±1.53 | +7.27±27.82 | NS |
| **PM oxidation** (pmol O2/s·mg) | 6.34±1.11 | 4.34±0.72 | -31.55±11.36 | NS |
| **GM oxidation** (pmol O2/s·mg) | 4.88±0.68 | 3.66±0.51 | -25.00±10.45 | NS |
| **ATP levels** (pmol ATP/mg protein) | 3.17±0.37 | 2.64±0.46 | -16.72±14.51 | NS |
| **Lipid peroxidation** (μM MDA+HAE/mg protein) | 1.12±0.12 | 1.22±0.12 | +8.93±16.96 | NS |

Values are presented as mean ± standard error of the mean and as a percentage of increase or decrease ± standard error of the mean. Case-control differences were sought by non-parametric statistical analysis.

ATP: adenosine triphosphate; Cell oxidation: cellular endogen oxidation (without substrates); CS: citrate synthase; GM oxidation: glutamate and malate oxidation; HAE: 4-hydroxyalkenal; IUGR: intrauterine growth restriction; MDA: malondialdehyde; NS: not significant; O_2_: oxygen; PBMC: peripheral blood mononuclear cells; PM oxidation: pyruvate and malate oxidation.
