# Supplementary material for: Mitochondrial implications in human pregnancies with intrauterine growth restriction and associated cardiac remodelling
Source: J Cell Mol Med. 2019 Apr 2;23(6):3962–73. doi: 10.1111/jcmm.14282 (PMC6533501; doi:10.1111/jcmm.14282)
Supplement: Supplementary file 3 [file JCMM-23-3962-s003.docx]

**Table S3.** **Experimental data in neonatal cord blood mononuclear cells of study groups.**

| ***Mitochondrial parameters in Neonatal CBMC*** | ***Control***  ***N = 22*** | ***IUGR***  ***N = 14*** | ***% of change*** | ***P value*** |
| --- | --- | --- | --- | --- |
| **Complex II** (nmol/minute·mg protein) | 37.26±5.00 | 40.01±10.45 | +7.38±28.05 | NS |
| **Complex II relative to CS activity** (nmol/minute·mg protein) | 0.43±0.04 | 0.73±0.17 | +69.77±39.53 | NS |
| **Complex IV** (nmol/minute·mg protein) | 21.09±1.89 | 17.14±3.17 | -18.73±15.03 | NS |
| **Complex IV relative to CS activity** (nmol/minute·mg protein) | 0.26±0.03 | 0.36±0.01 | +38.46±37.31 | NS |
| **Citrate Synthase** (nmol/minute·mg protein) | 86.06±6.79 | 52.33±10.85 | -39.19±12.61 | <0.05 |
| **Cell oxidation** (pmol O_2_/s·mg) | 4.58±0.74 | 2.49±0.65 | -45.63±14.19 | NS |
| **PM oxidation** (pmol O_2_/s·mg) | 4.83±1.22 | 2.42±0.55 | -49.90±11.39 | NS |
| **GM oxidation** (pmol O_2_/s·mg) | 3.93±1.02 | 1.74±0.45 | -55.73±11.45 | NS |
| **ATP levels** (pmol ATP/mg protein) | 1.15±0.17 | 1.85±0.53 | +60.87±46.09 | NS |
| **Lipid peroxidation** (μM MDA+HAE/mg protein) | 1.39±0.12 | 1.53±0.15 | +10.07±10.79 | NS |
| **Mitochondrial DNA depletion** (ratio mtDNA/nlDNA) | 101.09±10.25 | 95.41±8.15 | -5.62±8.06 | NS |

Values are presented as mean ± standard error of the mean and as a percentage of increase or decrease ± standard error of the mean. Case-control differences were sought by non-parametric statistical analysis.

ATP: adenosine triphosphate; CBMC: cord blood mononuclear cells; Cell oxidation: cellular endogen oxidation (without substrates); CS: citrate synthase; GM oxidation: glutamate and malate oxidation; HAE: 4-hydroxyalkenal; IUGR: intrauterine growth restriction; MDA: malondialdehyde; NS: not significant; O_2_: oxygen; PM oxidation: pyruvate and malate oxidation.
