# Supplementary material for: Mitochondrial implications in human pregnancies with intrauterine growth restriction and associated cardiac remodelling
Source: J Cell Mol Med. 2019 Apr 2;23(6):3962–73. doi: 10.1111/jcmm.14282 (PMC6533501; doi:10.1111/jcmm.14282)
Supplement: Supplementary file 4 [file JCMM-23-3962-s004.docx]

**Table S4. Significant associations between clinical data and experimental results in the cohort of IUGR and control pregnancies.**

| **Parameter** | **With respect to** | **Correlation coefficient** | **p** | **R^2^** |
| --- | --- | --- | --- | --- |
| **Birth weight** (g) | Placental weight (g) | 0.655 | 0.004 | 0.603 |
|  | BNP levels (pg/ml) | -0.600 | 0.000 | 0.526 |
|  | Oxygen consumption (PM oxidation)^a^ in placenta | 0.480 | 0.018 | 0.197 |
|  | Oxygen consumption (GM oxidation)^a^ in placenta | 0.505 | 0.008 | 0.279 |
|  | CI enzymatic activity^b^ in placenta | 0.412 | 0.026 | 0.195 |
|  | Sirtuin3/β-actin ratio (AU) in placenta | -0.470 | 0.008 | 0.224 |
| **Placental weight** (g) | Cord blood BNP levels (pg/ml) | -0.736 | 0.006 | 0.292 |
| **Cord blood BNP levels** (pg/ml) | CI enzymatic activity^b^ in placenta | -0.464 | 0.026 | 0.159 |
| **Oxygen consumption (GM oxidation)**^a^ **in maternal PBMC** | Oxygen consumption (GM oxidation)^a^ in neonatal CBMC | 0.544 | 0.007 | 0.115 |
| **CS activity in neonatal CBMC**^b^ | Oxygen consumption (Cell oxidation)^a^ in neonatal CBMC | 0.439 | 0.036 | 0.189 |
|  | Oxygen consumption (PM oxidation)^a^ in neonatal CBMC | 0.507 | 0.014 | 0.159 |
|  | Oxygen consumption (GM oxidation)^a^ in neonatal CBMC | 0.659 | 0.001 | 0.213 |
| **Oxygen consumption (GM oxidation)**^a^ **in neonatal CBMC** | Oxygen consumption (GM oxidation)^a^ in placenta | 0.466 | 0.033 | 0.383 |
| **Sirtuin3/β-actin ratio** (AU) **in placenta** | CI enzymatic activity^b^ in placenta | -0.416 | 0.034 | 0.095 |

Spearman correlations were used to seek for statistical associations. ^a^: pmol O2/s·mg protein; ^b^: nmol/minute·mg protein.

AU: arbitrary units; BNP: brain natriuretic peptide; CBMC: cord blood mononuclear cells; CI: MRC complex I; g: grams; GM oxidation: glutamate and malate oxidation; MRC: mitochondrial respiratory chain; PBMC: peripheral blood mononuclear cells; PM oxidation: pyruvate and malate oxidation; R^2^: coefficient of determination.
